# Supplementary material for: High-throughput sensitive screening of small molecule modulators of microexon alternative splicing using dual Nano and Firefly luciferase reporters
Source: Nat Commun. 2024 Jul 27;15:6328. doi: 10.1038/s41467-024-50399-6 (PMC11283458; doi:10.1038/s41467-024-50399-6)
Supplement: Supplementary file 1 — Supplementary Information [file 41467_2024_50399_MOESM1_ESM.pdf]

High-throughput sensitive screening of small molecule modulators of microexon alternative splicing using dual Nano and Firefly luciferase reporters

Supplementary Information

Supplementary Figure 1

A

Mef2d WT microexon (21nt): 5'-ACTGAGGACCATTTAGATCTG-3'
Mef2d V1 microexon (22nt): 5'-ACTGAGGACCATTTTAGATCTG-3'
Mef2d V2 microexon (20nt): 5'-ACTGAGGACCATTTAGATCG-3'

B

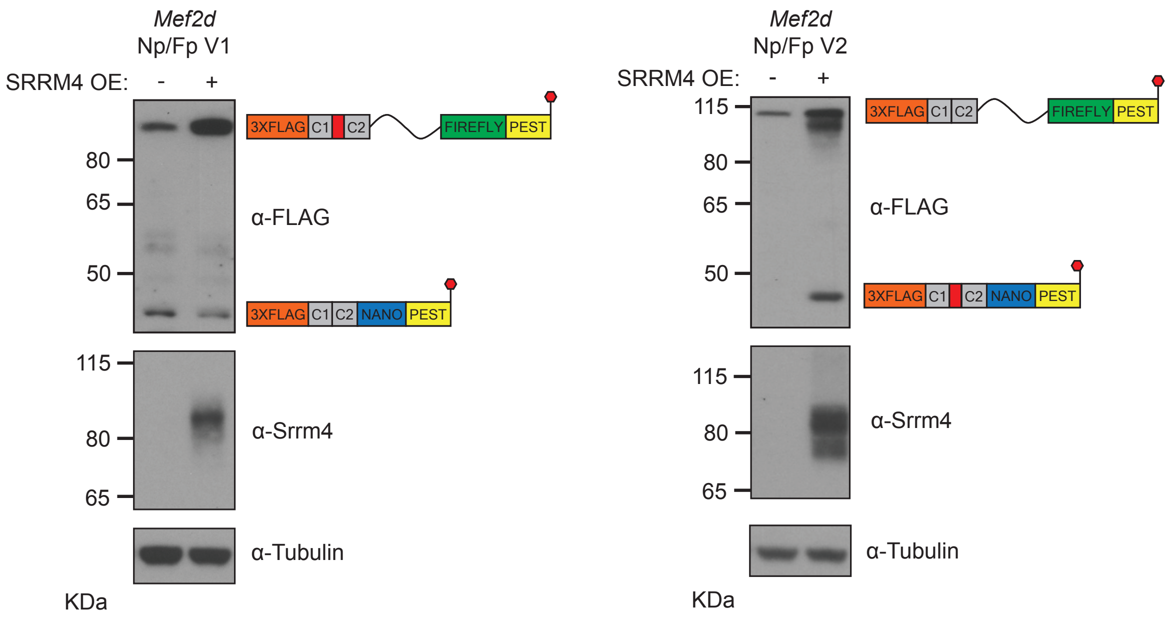

Supplementary Figure 1. Development of dual luciferase microexon splicing reporters and validation of response to increased expression of SRRM4.

(A) Sequences of modified Mef2d microexons in V1 and V2 reporters containing mutations that introduce a +1nt or -1nt shift in the downstream open reading frame, respectively.

(B) Western blotting using anti-FLAG antibody to detect changes in relative ratios of protein isoforms expressed from the Mef2d microexon splicing reporters in response to induced Srrm4 expression.

Supplementary Figure 2

A

Shank2 WT microexon (9nt): 5’-TGGACAAAG-3’
Shank2 V1 microexon (10nt): 5’-TGGACAAAAG-3’
Shank2 V2 microexon (8nt): 5’-TGGACAAG-3’

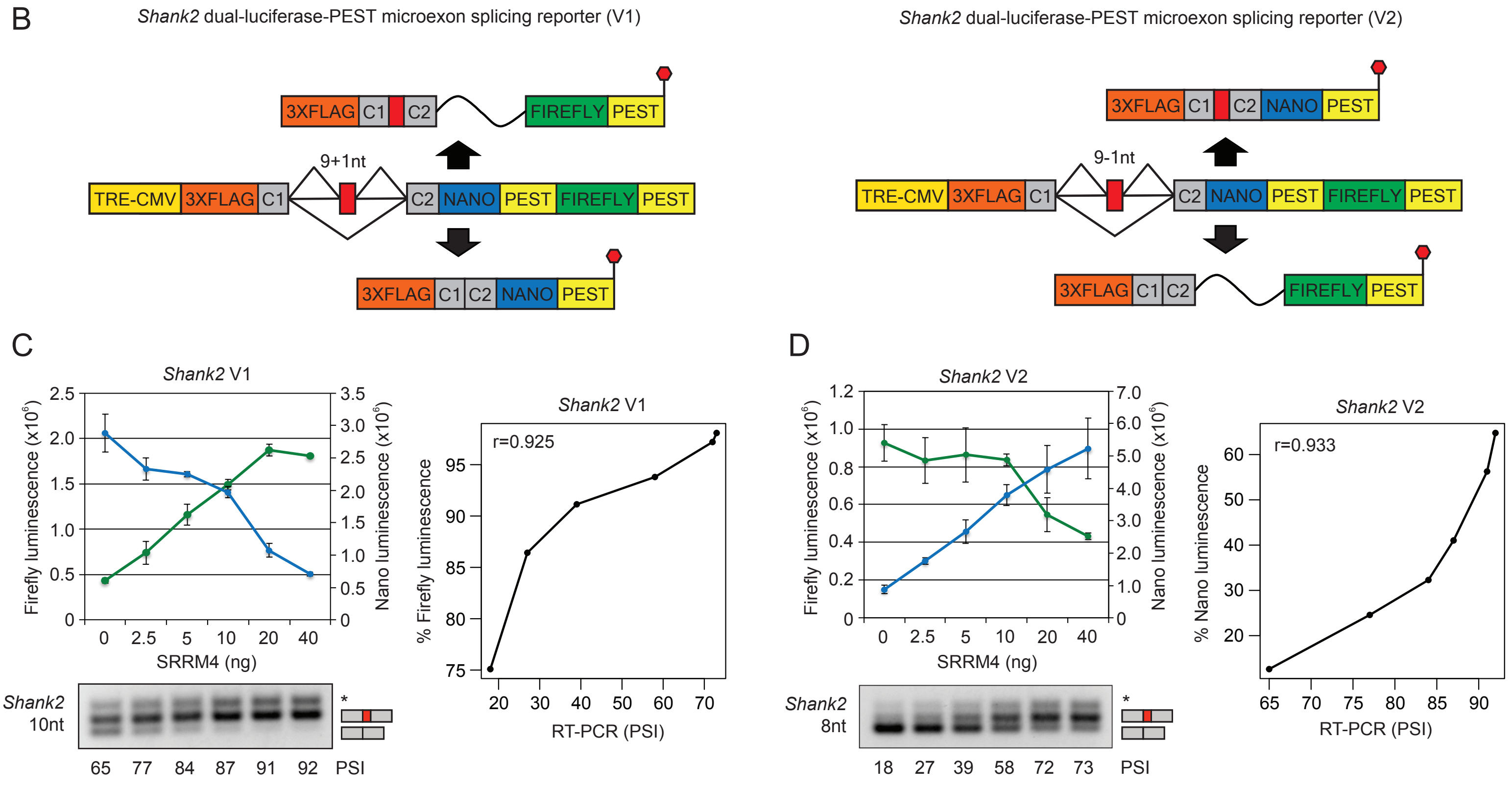

E

Shank2 V1 (9nt+1)

* 5’-GCCTCTCACTAGTGGACAAAAG-3’

5’-TGGACAAAAG-3’

Shank2 V2 (9nt-1)

* 5’-GCCTCTCACTAGTGGACAAG-3’

5’-TGGACAAG-3’

Supplementary Figure 2. Generation and validation of a Shank2 microexon dual-luciferase-PEST splicing reporters.

(A) Sequences of modified Shank2 microexons in V1 and V2 reporters containing mutations that introduce a +1nt or -1nt shift in the downstream open reading frame, respectively.

(B) Schematic diagram of reciprocal, dual-luciferase Shank2 microexon splicing reporters. Refer to Figure 1A legend for details.

(C) Quantification of Nano and Firefly luminescence from dual-luciferase Shank2 microexon splicing reporter V1 in response to transfection with increasing amounts of an SRRM4 expression vector (n=3, error bars = 1SD). RT-PCR validation of microexon percentage splicing inclusion (PSI) is shown in response to transfection with increasing amounts of SRRM4 expression vector, and correlation between precent Firefly luminescence and ΔPSI is indicated (r=0.925).

(D) Quantification of Nano and Firefly luminescence from dual-luciferase Shank2 microexon splicing reporter V2 characterized as in (B). Correlation between percent Nano luminescence and ΔPSI is indicated (r=0.933).

(E) Sequence of an additional spliced product (*) produced from the Shank2 microexon reporters as a consequence of usage of an in-frame alternative 3’ss.

Supplementary Figure 3

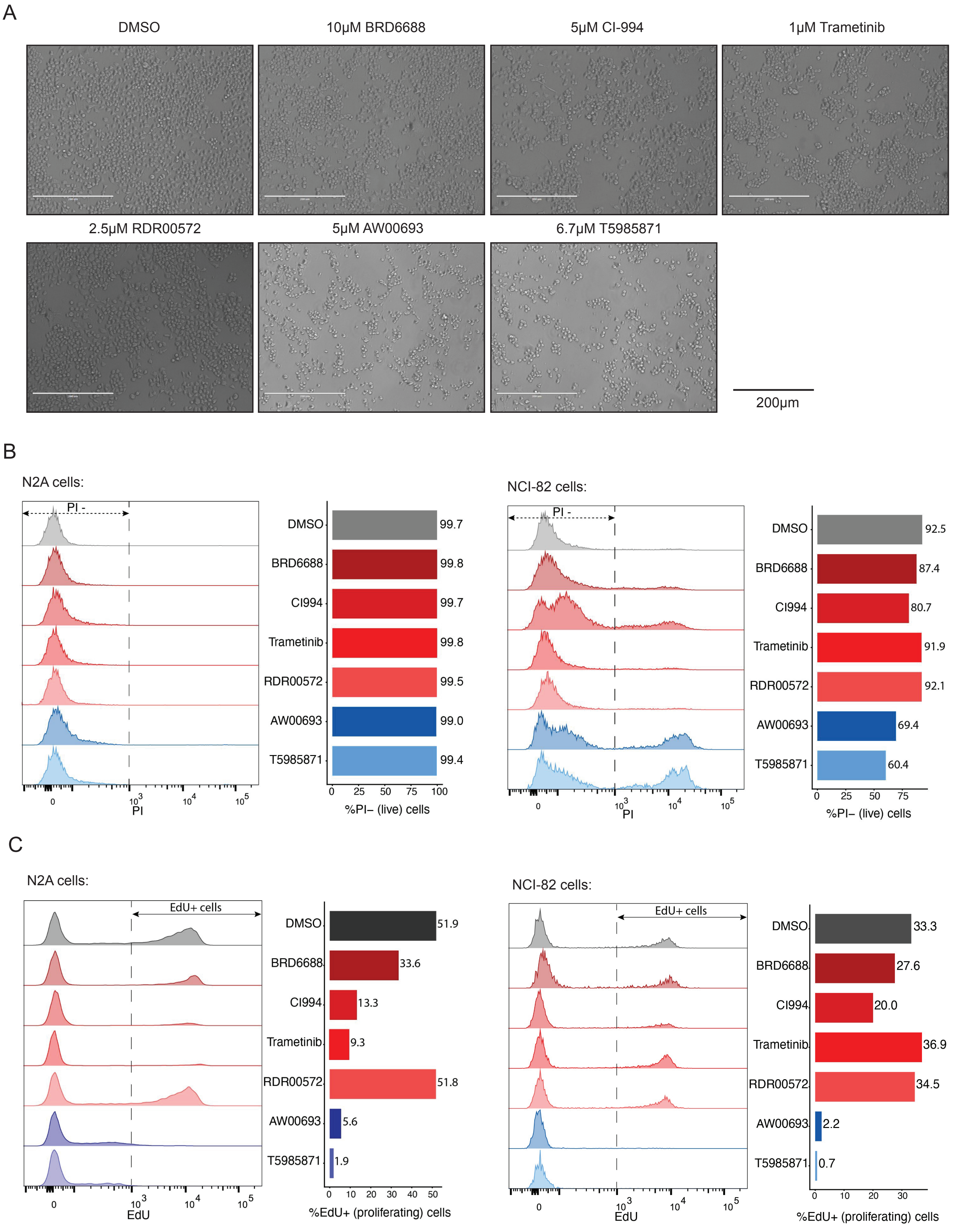

Supplementary Figure 3. Phenotypic characterization of N2A cells following treatment with compounds.

(A) Bright-field images of N2A cells after 24hrs treatment with compounds BRD6688, CI-994, RDR00572, Trametinib, AW00693 or T5985871.

(B) Cell viability analysis with Propidium iodide (PI) staining of N2A cells and NCI-82 cells after treatment with indicated compounds.

(C) Cell proliferation assay with Click-it EdU (5-ethynyl-2'-deoxyuridine) staining of N2A cells and NCI-82 cells following treatment with indicated compounds.

Supplementary Figure 4

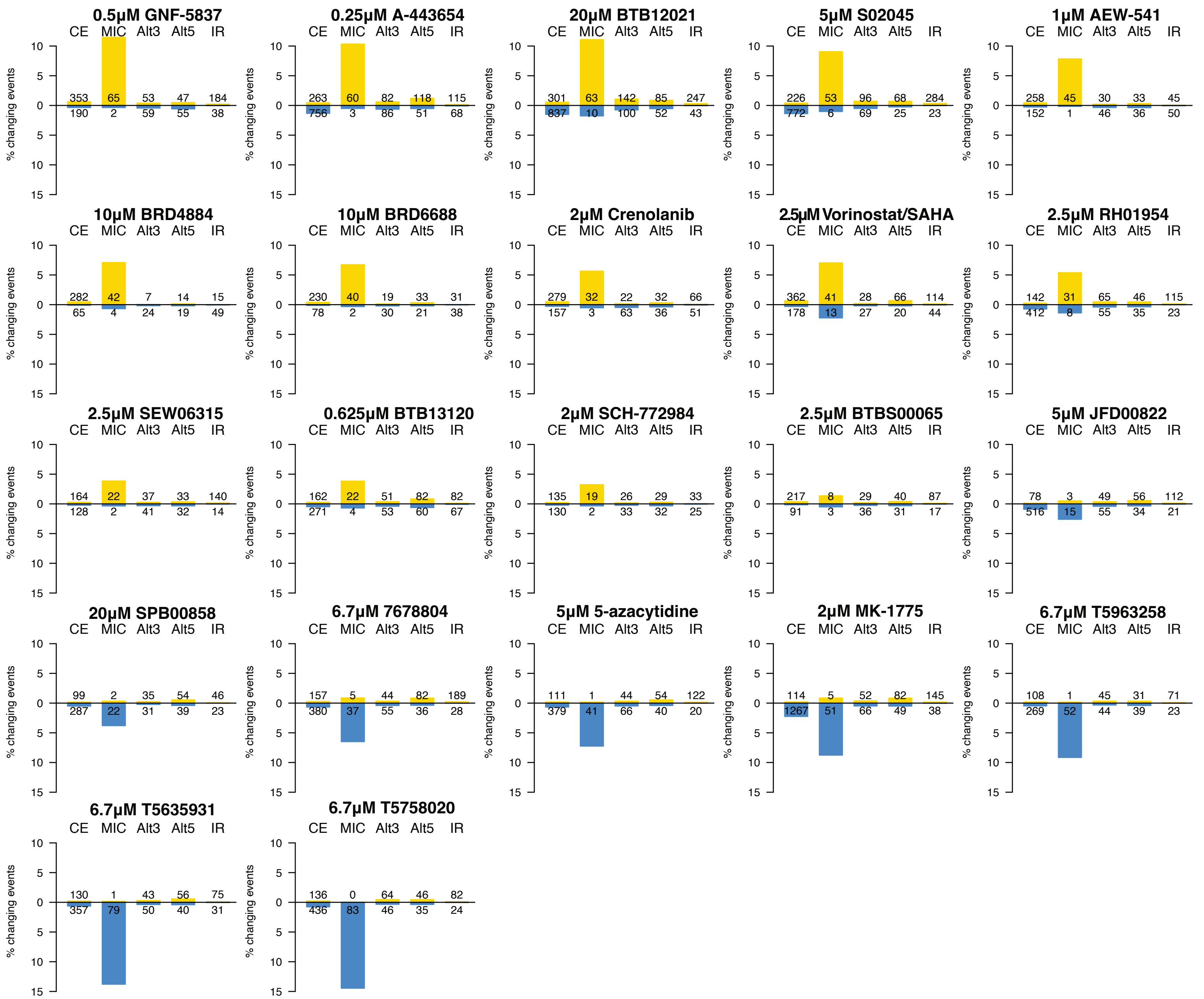

Supplementary Figure 4. Global changes in alternative splicing in N2A cells following 27 small molecule treatments.

Percentage of total detected alternative splicing events (all classes) with changing levels (≥10 ΔPSI) in N2A cells following treatments with 27 compounds. CE, cassette exons; MIC, microexons; Alt5/Alt3, alternative 5'/3'-splice sites; IR, intron retention. Absolute numbers of events with increased and decreased splicing levels are indicated.

Supplementary Figure 5

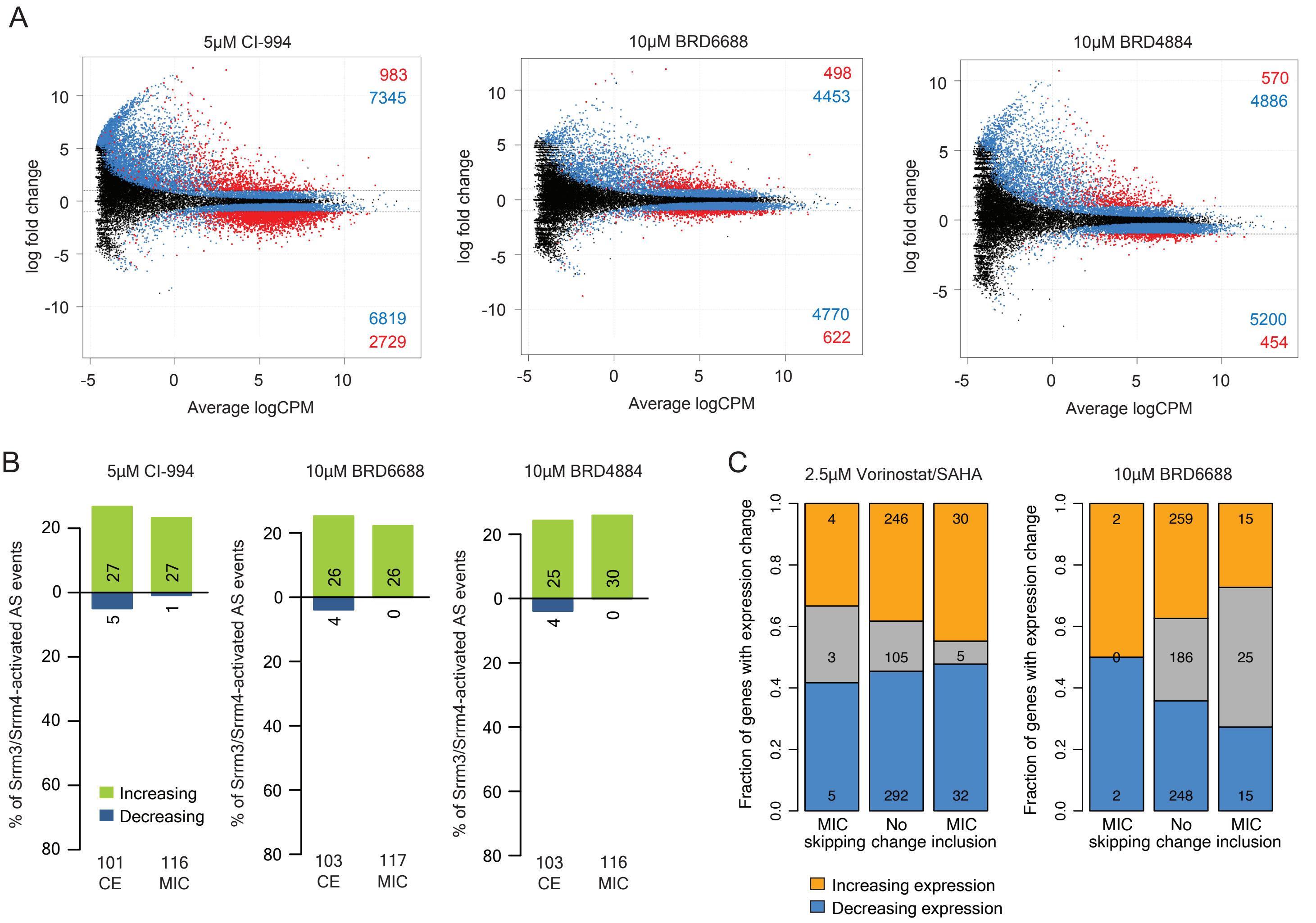

Supplementary Figure 5. Comparison of the transcriptome-wide effects of the Class I HDAC inhibitor CI-994 with the kinetically selective HDAC1-3 inhibitors BRD6688 & BRD4884.

(A) Global analysis of GE changes (logFC) following treatments with CI-994, BRD6688 and BRD4884 (FDR<0.05 (blue), FDR<0.05, LFC>1, maxRPKM>5 (red)).

(B) Percentage of alternative splicing events with changing levels (≥10 ΔPSI) among different classes of Srrm4/3-regulated alternative splicing events (CE, MIC, Alt3, Alt5 and IR) following treatments with CI-994, BRD6688 and BRD4884.

(C) Fraction of genes containing microexons (MIC) with increased skipping, no change, or increased inclusion, with an increase, no change, or decreases in expression upon treatment with Vorinostat or BRD6688.

Supplementary Figure 6. RT-PCR validation of compound treatments using cells depleted of Srrm4, and RT-PCR validation of combined compound treatments.

(A) RT-PCR analysis of splicing changes following compound treatments of N2A cells expressing either a control shRNA targeting GFP, or shRNA targeting Srrm4 (gel images representative of three replicate experiments).

(B) RT-PCR validations of splicing level changes (ΔPSI) in N2A cells following single treatments with the drug-like molecule RDR00572 or a single HDAC inhibitor (CNS-26, BRD6688, CI-994 or MS-275), or combined treatment with a HDAC inhibitor and RDR00572. Data generated from single samples. The final concentration of small molecules (in single or combined treatments) is 5μM. PSI was determined for individual microexons from the following genes; Clasp2 (blue), L1cam (red), Pus7 (orange), Snx14 (green). Mean ΔPSI for all tested microexons is shown in grey bar plots.

Supplementary Figure 6

A

B

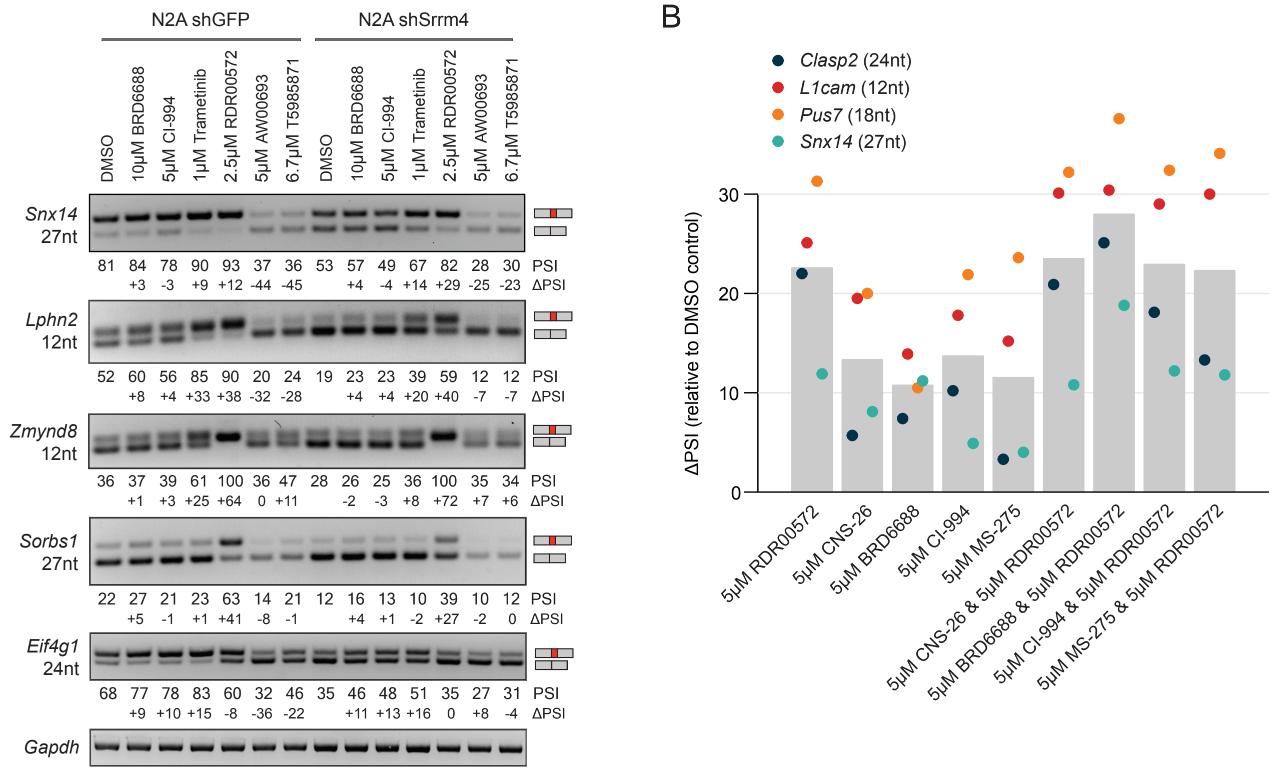

Supplementary Table 1. Small molecule screening data

Category	Parameter	Description
Assay	Type of assay	Dual-luciferase splicing reporters
	Target	*Mef2d* microexon
	Primary measurement	Luminescence from Nano luciferase and Firefly luciferase
	Key reagents	Nano-Glo Dual-Luciferase Reporter Assay System (Promega, N1610)
	Assay protocol	Doxycycline-inducible N2A Flp-In rtTA3 stable cell lines were seeded at 2,000 cells per well in 50μL medium in 384-well plates (Biomek FX Laboratory Automation Workstation, Beckman Coulter). 200nL of compounds and DMSO controls were added using a dedicated pin tool (Multimek, Beckman) to achieve final concentrations of 4μM (except for the PKI library, which was screened at a final concentration of 0.5μM), for in total 24-hour treatment. Six hours post compound treatments, 10μL of 6μg/mL doxycyline was added (1μg/mL final concentration) to induce the expression of dual-luciferasePEST splicing reporters. Luminescence was detected using the Nano-Glo Dual-Luciferase Reporter Assay System (Promega, N1610) and quantified using a plate reader (Envision, Perkin Elmer).
	Additional comments	
Library	Library size	~95,000
	Library composition	A diverse collection of approximately 95,000 small molecules, consisting of ~89,000 synthetic drug-like screening compounds and ~6,000 bioactive drugs were assembled for high-throughput screening. The libraries included ~12,000 drug-like small molecules from the Chembridge Diversity Set, ~52,000 drug-like small molecules from the Maybridge HitDiscover collection, ~24,000 small molecules from a 'PAIN-free' collection curated from Enamine, ChemDiv and Chembridge by the Ontario Institute for Cancer Research (OICR), as well as approximately ~6,000 bioactive molecules assembled from several smaller libraries, including TOCRIS, LOPAC, Prestwick and a PKI library.
	Source	Lunenfeld-Tanebaum Research Institute (LTRI) Network Biology Collaborative Centre
	Additional comments	
Screen	Format	384-well
	Concentration(s) tested	4μM or 0.5μM
	Plate controls	Negative controls: 0.4% DMSO, Positive controls: 200nM TSA, 150nM TSA, 40nM TSA or 4μM Scriptaid.
	Reagent/ compound dispensing system	Biomek FX Laboratory Automation Workstation, (Beckman Coulter) and Multimek (Beckman Coulter).
	Detection instrument and software	Envision, Perkin Elmer
	Assay validation/QC	
	Correction factors	
	Normalization	B-scores (see Methods for details).
	Additional comments	
Post-HTS analysis	Hit criteria	Custom hyperbolae (see Methods for details).
	Hit rate	Putative activators: 0.8%, putative inhibitors: 0.7%
	Additional assay(s)	Independent serial dilutions, and RT-PCR validation
	Confirmation of hit purity and structure	Compounds were repurchased from molport.com

Additional comments
