# Supplementary material for: High-throughput sensitive screening of small molecule modulators of microexon alternative splicing using dual Nano and Firefly luciferase reporters
Source: Nat Commun. 2024 Jul 27;15:6328. doi: 10.1038/s41467-024-50399-6 (PMC11283458; doi:10.1038/s41467-024-50399-6)
Supplement: Supplementary file 3 — Description of Additional Supplementary Files [file 41467_2024_50399_MOESM3_ESM.pdf]

Description of Additional Supplementary Files

Supplementary Data 1. Luminescence data from primary screen of ~95,000 small molecules. F1 = Firefly luminescence for V1 reporter, N1 = Nano luminescence for V1 reporter, F2 = Firefly luminescence for V2 reporter, N2 = Nano luminescence for V2 reporter. F1/N1 B-score = B-score of the Firefly/Nano luminescence ratio for V1 reporter, N2/F2 B-score = Bscore of the Nano/Firefly luminescence ratio for V2 reporter.

Supplementary Data 2. Information for 576 compounds tested in serial dilution assay.

Supplementary Data 3. RT-PCR validation data for 91 compounds tested by RT-PCR. ΔPSI calculated relative to 0.4% DMSO controls. Ranking of putative activators and putative inhibitors calculated based on mean ΔPSI for Srrm4-dependent events.

Supplementary Data 4. Information for 27 compounds characterized by RNA-seq analysis.
