# Supplementary material for: High-throughput sensitive screening of small molecule modulators of microexon alternative splicing using dual Nano and Firefly luciferase reporters
Source: Nat Commun. 2024 Jul 27;15:6328. doi: 10.1038/s41467-024-50399-6 (PMC11283458; doi:10.1038/s41467-024-50399-6)
Supplement: Supplementary file 8 — Reporting Summary [file 41467_2024_50399_MOESM8_ESM.pdf]

nature portfolio

Corresponding author(s): Andrew J Best & Benjamin J Blencowe

Last updated by author(s): Jun 9, 2024

Reporting Summary

Nature Portfolio wishes to improve the reproducibility of the work that we publish. This form provides structure for consistency and transparency in reporting. For further information on Nature Portfolio policies, see our Editorial Policies and the Editorial Policy Checklist.

Statistics

For all statistical analyses, confirm that the following items are present in the figure legend, table legend, main text, or Methods section.

n/a	Confirmed	
☐	☒	The exact sample size (*n*) for each experimental group/condition, given as a discrete number and unit of measurement
☐	☒	A statement on whether measurements were taken from distinct samples or whether the same sample was measured repeatedly
☐	☒	The statistical test(s) used AND whether they are one- or two-sided<br>*Only common tests should be described solely by name; describe more complex techniques in the Methods section.*
☐	☒	A description of all covariates tested
☐	☒	A description of any assumptions or corrections, such as tests of normality and adjustment for multiple comparisons
☐	☒	A full description of the statistical parameters including central tendency (e.g. means) or other basic estimates (e.g. regression coefficient) AND variation (e.g. standard deviation) or associated estimates of uncertainty (e.g. confidence intervals)
☐	☒	For null hypothesis testing, the test statistic (e.g. *F*, *t*, *r*) with confidence intervals, effect sizes, degrees of freedom and *P* value noted<br>*Give P values as exact values whenever suitable.*
☒	☐	For Bayesian analysis, information on the choice of priors and Markov chain Monte Carlo settings
☒	☐	For hierarchical and complex designs, identification of the appropriate level for tests and full reporting of outcomes
☐	☒	Estimates of effect sizes (e.g. Cohen's *d*, Pearson's *r*), indicating how they were calculated

Our web collection on statistics for biologists contains articles on many of the points above.

Software and code

Policy information about availability of computer code

Data collection	RNA-Seq data were processed using the vast-tools pipeline, version 2.1 https://github.com/vastgroup/vast-tools. GE changes were analyzed by pseudo-aligning pre-trimmed reads to GENCODE vM21 transcripts using Salmon v0.14.1. GO enrichment analysis for genes containing alternative splicing changes was performed with FuncAssociate 3.0.
Data analysis	Custom R code was generated to normalize raw luminescence data and call hits as described in the Methods. Differential gene expression was analyzed using Salmon 0.14.1 and the R packages tximport v1.9.7 and edgeR v3.32. Alternative splicing analysis was conducted with vast-tools v2.1. R scripts used to normalize RNA-Seq data for differential expression and splicing analysis is available on GitHub at https://github.com/UBrau/ProcessDE (v0.5.1) Ref: https://github.com/UBrau/ProcessDE; DOI: 10.5281/zenodo.10265546 and https://github.com/UBrau/ProcessVast (v0.5.0) Ref: https://github.com/UBrau/ProcessVast; DOI: 10.5281/zenodo.10407129, respectively.

For manuscripts utilizing custom algorithms or software that are central to the research but not yet described in published literature, software must be made available to editors and reviewers. We strongly encourage code deposition in a community repository (e.g. GitHub). See the Nature Portfolio guidelines for submitting code & software for further information.

Data

Policy information about availability of data

All manuscripts must include a data availability statement. This statement should provide the following information, where applicable:

- Accession codes, unique identifiers, or web links for publicly available datasets
- A description of any restrictions on data availability
- For clinical datasets or third party data, please ensure that the statement adheres to our policy

Raw luminescence data and B-score ratios from the primary and secondary screens are available as supplementary information. RNA-Seq data generated in the course of this study are available on GEO as series GSE228599.

Research involving human participants, their data, or biological material

Policy information about studies with human participants or human data. See also policy information about sex, gender (identity/presentation), and sexual orientation and race, ethnicity and racism.

Reporting on sex and gender	N/A
Reporting on race, ethnicity, or other socially relevant groupings	N/A
Population characteristics	N/A
Recruitment	N/A
Ethics oversight	N/A

Note that full information on the approval of the study protocol must also be provided in the manuscript.

Field-specific reporting

Please select the one below that is the best fit for your research. If you are not sure, read the appropriate sections before making your selection.

☒ Life sciences ☐ Behavioural & social sciences ☐ Ecological, evolutionary & environmental sciences

For a reference copy of the document with all sections, see nature.com/documents/nr-reporting-summary-flat.pdf

Life sciences study design

All studies must disclose on these points even when the disclosure is negative.

Sample size	Experiments were generally performed with a minimum of two biological replicates per sample, or else validated across multiple cell lines or analyzed alongside multiple structurally related compounds in parallel. For the primary screen, compounds were screened with two independent reciprocal reporters in order to minimize false positives. RNA-seq analysis was performed with 2 biological replicates for the 0.4% DMSO controls and HDACi treatments, or from 1 biological sample for all other compounds tested, in order to maximize the number of compounds analyzed via RNA-seq. In vivo treatments were administered prior to obtaining the genotype of each mice, and sufficient treatments were performed in order to generate a minimum of two mice per genotype and treatment.
Data exclusions	No data was excluded from the analyses in this study.
Replication	The experimental findings from the primary screen (dual-luciferase reporters) were independently validated by re-picking compounds and performing serial dilutions with 2 biological replicates. The hits validated via serial dilution were subsequently independently validated by performing RT-PCR analysis from independent biological samples. Several compounds were re-purchased from an independent supplier (Molport.com) and validated across multiple cell lines , or in vivo.
Randomization	N/A
Blinding	Investigators were blind to the identity of drug treatments during data collection and analysis. Investigators were also blind to the genotype of mice when administrating subcutaneous injections.

Reporting for specific materials, systems and methods

We require information from authors about some types of materials, experimental systems and methods used in many studies. Here, indicate whether each material, system or method listed is relevant to your study. If you are not sure if a list item applies to your research, read the appropriate section before selecting a response.

Materials & experimental systems

n/a	Involved in the study	
☐	☒	Antibodies
☐	☒	Eukaryotic cell lines
☒	☐	Palaeontology and archaeology
☐	☒	Animals and other organisms
☒	☐	Clinical data
☒	☐	Dual use research of concern
☒	☐	Plants

Methods

n/a	Involved in the study	
☒	☐	ChIP-seq
☒	☐	Flow cytometry
☒	☐	MRI-based neuroimaging

Antibodies

Antibodies used	anti-a-Tubulin (Sigma-Aldrich T6074) at 1:5000; mouse anti-PTBP1 (Invitrogen 32-4800) at 1:1000; mouse anti-QKI (EMD Millipore MABN624) at 1:500; rabbit anti-Rbfox2 (Bethyl A300-864A-T) at 1:1000; Rabbit anti-Srsf11 (Thermo Fisher PA5-37056) at 1:1000; Rabbit anti-Srrm4 (Calarco et al., 2009) at 1:5000; Rabbit anti-Rbm38 (Abcam ab200403) at 1:1000.
Validation	For information on validation of the rabbit anti-Srrm4 primary antibody, please refer to Calarco et al., Cell, 2009.<br>For information on validation of commercially available primary antibodies, please refer to the manufacturer's website.

Eukaryotic cell lines

Policy information about cell lines and Sex and Gender in Research

Cell line source(s)	All cell lines used in this study were sourced from ATCC.
Authentication	The cell lines used in this study were not independently authenticated.
Mycoplasma contamination	All cell lines tested negative for mycoplasma contamination.
Commonly misidentified lines (See ICLAC register)	No commonly misidentified cell lines were used in the study.

Animals and other research organisms

Policy information about studies involving animals; ARRIVE guidelines recommended for reporting animal research, and Sex and Gender in Research

Laboratory animals	All mice used in this study were C57BL/6. All drug treatments were performed on wild-type and heterozygous mice generated from nSR100+/Δ7-8 inter-crosses and fostered by nSR100+/Δ7-8 females. Beginning at postnatal day 2, wild-type or heterozygous pups received a daily subcutaneous injection consisting of a mock treatment (75% Physiological Saline, 20% Kolliphor EL and 5% DMSO) or a 30mg/kg dose of RDR00572 (75% Physiological Saline, 20% Kolliphor EL and 5% DMSO/RDR00572). Each pup received 7 daily injections between P2-P8.
Wild animals	The study did not involve wild animals.
Reporting on sex	Sex was not considered in this study.
Field-collected samples	This study did not involve samples collected from the field.
Ethics oversight	All experiments were conducted in compliance with the Animals for Research Act of Ontario and the Guidelines of the Canadian Council on Animal Care. The Centre for Phenogenomics (TCP) Animal Care Committee reviewed and approved all procedures conducted on animals at TCP. To minimize stress to animals, mice were housed with a 12-hour light/12-hour dark cycle, with an ambient temperature between 65-75°F with 40-60% humidity.

Note that full information on the approval of the study protocol must also be provided in the manuscript.

Plants

Seed stocks	*Report on the source of all seed stocks or other plant material used. If applicable, state the seed stock centre and catalogue number. If plant specimens were collected from the field, describe the collection location, date and sampling procedures.*
Novel plant genotypes	*Describe the methods by which all novel plant genotypes were produced. This includes those generated by transgenic approaches, gene editing, chemical/radiation-based mutagenesis and hybridization. For transgenic lines, describe the transformation method, the number of independent lines analyzed and the generation upon which experiments were performed. For gene-edited lines, describe the editor used, the endogenous sequence targeted for editing, the targeting guide RNA sequence (if applicable) and how the editor was applied.*
Authentication	*Describe any authentication procedures for each seed stock used or novel genotype generated. Describe any experiments used to assess the effect of a mutation and, where applicable, how potential secondary effects (e.g. second site T-DNA insertions, mosiacism, off-target gene editing) were examined.*
